# Natural environment promotes deeper brain functional connectivity than built environment

**DOI:** 10.1186/1471-2202-16-S1-P294

**Published:** 2015-12-18

**Authors:** Zheng Chen, Yujia He, Yuguo Yu

**Affiliations:** 1 Department of Landscape Studies, College of Architecture and Urban Planning, Tongji University, Shanghai, 200092; China; 2 The State Key Laboratory of Medical Neurobiology and Institutes of Brain Science, School of Life Sciences, Fudan University, Shanghai, 200433;China

## 

Not only genes but also living environments can effectively shape living human infant brain growth and function performance through learning-driven neural plasticity. However, few evidences demonstrated that exposure to different environments may modulate adult brain cognitive functions [1]. This study examined this issue by using the Emotiv EPOC wireless EEG headset [2] and accompanying software. Brain waves are measured in terms of amplitude (10-100 microvolts) and five frequency bands, i.e., δ (0.5-4 Hz), θ (4-8 Hz), α (8-12 Hz), β (13-25 Hz) and γ (25-70), are examined. Sixteen college students were recruited and randomly assigned to the two conditions. Participants were asked to sit either in a built environment (i.e., a traffic island under an elevated highway), or in a natural environment (i.e., a heavily wooded campus garden). They were first sitting facing walls as baselines excluded for visual exposure for eight one-minute sessions with their eye open and closed in turns (OCOCOCOC), and then they turned to scene facing and exposed to the environment for 20 minutes. EEG was measured in the latter 10 minutes of exposure, as well as during eye-open and eye-closed baseline sessions.

**Figure 1:**
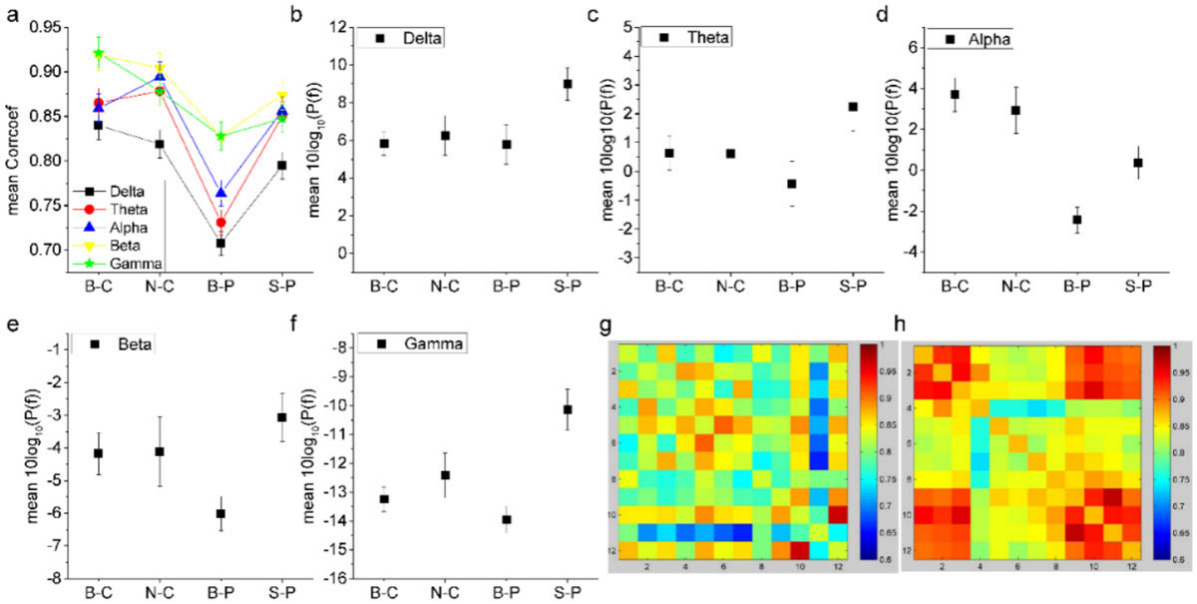
**Results of the exposure to built environment (B) and that to natural environment (N), were reported in mean power of δ band (b), θ band (c), α band (d), β band (e) and γ band (f)**. Average Cross-correlations of the 12 channels were displayed in (a). Cross-correlations of mean β powers between channels for to built environment (g) and natural environment (h) ,respectively. Wall-facing eye-closed pretest baselines were marked as -C, while post-exposure statuses were marked as -P.

Functional connectivity analysis revealed that subjects with eye close in both environments have stronger or deeper functional connectivity among different brain regions than eye-opened cases, while eye-opened subjects walking in natural environments have stronger functional connectivity than in highway environments (see Figure 1a). Interestingly, power spectrum analysis showed that EEG powers in all the frequency bands are higher in natural environment than in built environment (see Figure 1b~1f), indicating large-amplitude synchronized EEG waves in the brains of natural environment which strengthen deeper functional connectivities among brain regions (see Figure 1g,h for example). These results suggest that natural environment may promote a better brain performance than built environment.
